# Choroidal thickness measured using swept-source optical coherence tomography is reduced in patients with type 2 diabetes

**DOI:** 10.1371/journal.pone.0191977

**Published:** 2018-02-02

**Authors:** Beatriz Abadia, Ines Suñen, Pilar Calvo, Francisco Bartol, Guayente Verdes, Antonio Ferreras

**Affiliations:** 1 IIS-Aragon, Department of Ophthalmology, Miguel Servet University Hospital, Zaragoza, Spain; 2 Department of Surgery, Gynecology and Obstetrics, University of Zaragoza, Zaragoza, Spain; 3 Department of Endocrinology, Hospital de Alcañiz, Teruel, Spain; Weill Cornell Medical College in Qatar, QATAR

## Abstract

**Objective:**

To compare choroidal thickness between patients with type 2 diabetes (T2D) and healthy controls measured using swept-source optical coherence tomography (SS-OCT).

**Methods:**

The sample comprised 157 eyes of 94 T2D patients, 48 eyes of which had diabetic macular edema (DME), and 71 normal eyes of 38 healthy patients. Subfoveal (SF) choroidal thickness, and choroidal thickness at 500-μm intervals up to 2500 μm nasal and temporal from the fovea were measured using the SS-OCT. Choroidal thicknesses were compared between groups using Student’s t-test. Additionally, Pearson correlations were calculated between diabetes duration, glycosylated hemoglobin (HbA1c) levels, and choroidal thickness.

**Results:**

Mean diabetes duration was 16.6±9.5 years, while mean glycosylated hemoglobin was 7.7±1.3%. Overall, the choroid was significantly thinner in T2D patients. Individuals with DME had reduced choroidal thickness in all measurements, except at 2000 and 2500-μm nasal positions, compared to healthy controls. There was a moderate correlation between choroidal thickness and HbA1c levels in DME patients (SF: r = 0.342; p = 0.017). Diabetes duration did not correlate significantly with choroidal thickness.

**Conclusion:**

SS-OCT measurements revealed that the choroid was significantly thinner in T2D patients, moderate non-proliferative diabetic retinopathy patients, and DME patients than in healthy individuals. Further studies are needed to clarify the effect of diabetes on this layer and the relationship between choroidal thickness and DME.

## Introduction

Diabetes mellitus (DM) is chronic disease affecting 415 million people worldwide, and the prevalence is expected to rise to an estimated 642 million by the year 2040 [1]. The choroidal layer supplies blood to the outer layers of the retina and may play a key role in the pathophysiologic mechanism of diabetic retinopathy (DR). The most consequential changes of the choroid mainly affect the choriocapillaris layer, but may also extend to larger vessels located in the outer choroidal layers [2,3]. The choroid seems to play a role in different retinal pathologies [4]. A better understanding of the morphology and function of this vascular structure could facilitate the management of DR [5]. Recent studies regarding neovascular age-related macular degeneration and diabetes reported that choroidal thickness may predict the response to antiangiogenic agents [5,6]. Consequently, the assessment of choroidal changes may help to better make therapeutic decisions and to improve treatment follow-up.

Before the introduction of swept-source optical coherence tomography (SS-OCT) in clinical practice, choroidal thickness was evaluated by enhanced-depth imaging spectral domain (SD)-OCT [7–10]. Nevertheless, SS-OCT allows for faster scanning speed and its longer wavelength enables deeper penetration in the choroid to reveal more details and a clearer sclero-choroidal interface [11–13]. Consequently, the higher contrast of the images acquired with SS-OCT may lead to a better layer segmentation and more accurate measurements.

The purpose of the present study was to prospectively analyze the choroidal thickness measured by SS-OCT in patients with type 2 diabetes (T2D) having different degrees of DR with or without diabetic macular edema (DME) and compare them to age-matched healthy controls.

## Materials and methods

### Patient eligibility

This study adhered to the tenets of the Declaration of Helsinki and was approved by the Clinical Research Ethics Committee of Aragón (CEICA). Study-naïve patients with T2D were recruited from the Retina Unit of Miguel Servet University Hospital at Zaragoza (Spain) and control patients were selected from among healthy volunteers. All white individuals from December 2015 to July 2016 who met the inclusion criteria were consecutively pre-enrolled. Five patients with T2D did not provide informed consent, and were excluded from further analysis.

Participants were eligible if they were older than 18 years of age, with a refractive error of less than 6 spherical diopters and/or 2 diopters cylinder, axial length (AL) ≤26 mm, and euthyroid. Exclusion criteria included opacity of the optical media that could interfere with the quality of the OCT (signal/noise ratio <70/100), previous treatment with focal laser photocoagulation, panretinal photocoagulation, intravitreal anti-vascular endothelial growth factor or steroid injections, previous treatment with potentially toxic drugs to the retina and/or optic nerve, eye diseases that could affect retinal or choroidal anatomy, inflammatory diseases or active or recent infection (ocular and/or systemic), systemic treatment with corticosteroids, immunosuppressive drugs or biologic therapies, pregnancy, and puerperium.

Participants underwent full ophthalmologic examination: clinical history, including duration of diabetes in T2D patients; best-corrected visual acuity (BCVA, decimal scale), biomicroscopy of the anterior segment using a slit lamp, Goldmann applanation tonometry, and ophthalmoscopy of the posterior segment, and AL measured using optical biometry (IOL Master Zeiss; Jena, Germany). In all participants, glycosylated hemoglobin (HbA1c) was also measured.

### Diabetic retinopathy grading

Study-naïve patients with T2D were diagnosed based on the criteria of the American Diabetes Association and all were negative for anti-glutamic acid decarboxylase antibody. This group was divided into five subgroups depending on the degree of DR according to the Early Treatment Diabetic Retinopathy Study (ETDRS) criteria [14]: no DR, mild non-proliferative DR (NPDR), moderate NPDR, severe NPDR, and proliferative DR (PDR). DME was assessed by clinical examination and SS-OCT imaging.

### Choroidal thickness measurements using SS-OCT

**Fig 1:**
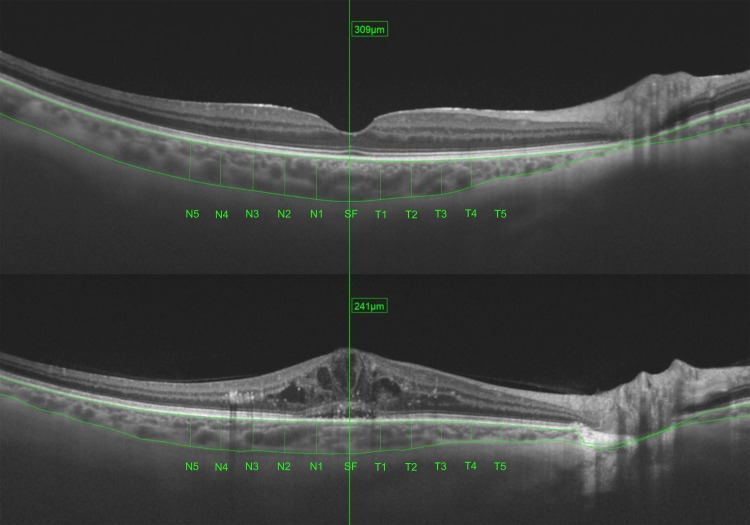
Choroidal measurements at subfoveal, nasal (N1, N2, N3, N4, and N5), and temporal (T1, T2, T3, T4, and T5) locations. Measurements were acquired at 500-μm intervals up to 2500 μm nasal and temporal to the fovea. SS-OCT images of representative cases from a healthy participant (top image) and a DME patient (bottom image).

Each SS-OCT (3D deep range imaging [DRI] OCT Triton [plus], Topcon Corporation, Tokyo, Japan) scan comprised a horizontal line of 12 mm centered on the fovea and was obtained by an experienced technician. OCT scans were performed at the same time in all patients: between 4:00 pm and 7:00 pm. The choroidal layer was automatically segmented using the proprietary algorithm from the outer edge of the hyper-reflective retinal pigment epithelial line to the inner edge of the sclera. DRI SS-OCT Triton images were directly visualized by an independent observer to detect automated segmentation errors of the choroidal layer. After automatic delineation of the choroid, thickness measurements were obtained manually at 11 positions using a caliper: five measurements nasal (N1, N2, N3, N4, and N5) and temporal (T1, T2, T3, T4, and T5) to the fovea were taken at 500-μm intervals along with the subfoveal (SF) measurement (Fig 1). Automatic segmentation errors were recorded and corrected manually. Scans with a lower quality (<70/100) were discarded (S1 Database).

### Statistical analysis

Statistical analyses were performed using IBM SPSS (version 23.0; IBM Corporation, Somers, NY, USA) and MedCalc (version 12; MedCalc Software, Mariakerke, Belgium) statistical software. A sample size calculation estimated that 45 eyes would be necessary for a type 1 error rate of 0.05 and a power of 80% to detect a mean difference of 10%, assuming that mean choroidal thickness was 260.8±60.9 μm (analysis performed with MedCalc software version 12; Mariakerke, Belgium) [15]. All the variables followed a normal distribution as verified by the Kolmogorov-Smirnov test. Student’s t-test or ANOVA (Scheffe test for post-hoc analysis) were used to compare choroidal thicknesses between groups. Differences between percentages were assessed by the chi-square test. Additionally, Pearson correlations were calculated between diabetes duration, HbA1c levels, and choroidal thickness. For all analyses, p<0.05 was considered statistically significant.

## Results

### Demographics and clinical characteristics

A total of 228 eyes of 132 patients were included in the study (50.9% women, 50.9% right eyes, mean age 67.6±8.1 years, range 49–86 years). Mean HbA1c was 5.6±0.3% in the healthy group and 7.7±1.3% in the T2D patients. Mean DM duration in T2D patients was 16.6±9.5 years. Mean OCT scan quality was 93.9/100±4.4, while an automatic and accurate segmentation was achieved in 82.9% of cases. The healthy group included 71 eyes and the T2D group comprised 157 eyes (48 eyes had DME). Based on the DR severity scale, the T2D group had 49 eyes without DR, 27 eyes with mild NPDR, 60 eyes with moderate NPDR, 14 eyes with severe NPDR, and 7 with PDR. The characteristics of each group are summarized in Table 1.

**Table 1:** Characteristics of the study sample. Age, BCVA, IOP, Spherical equivalent, Axial length, DM duration, HbA1c, Triglycerides, HDL cholesterol, LDL cholesterol, Systolic pressure, Diastolic pressure, and Scan quality are expressed as mean ± standard deviation.

	Healthy	No DR	Mild NPDR	Moderate NPDR	Severe NPDR	PDR
Age (y)	68 ± 8.4	66.2 ± 8.9	68.5 ± 7.0	68.3 ± 7.5	68.9 ± 8	59.8 ± 5.1
BCVA (decimal)	0.8 ± 0.1	0.8 ± 0.1	0.7 ± 0.2	0.6 ± 0.2	0.5 ± 0.2	0.5 ± 0.1
BCVA (logMAR)	0.06 ± 0.1	0.08 ± 0.1	0.14 ± 0.2	0.18 ± 0.2	0.33 ± 0.3	0.27 ± 0.1
IOP (mmHg)	16 ± 2.3	17.2 ± 3.3	15.9 ± 3.2	16.9 ± 3.2	17 ± 2.3	16.8 ± 3.0
Spherical equivalent (diopters)	-0.9 ± 2.2	-0.3 ± 1.7	-0.2 ± 1.7	-0.2 ± 0.7	-0.6 ± 2.2	-0.4 ± 1.0
Axial length (mm)	23.9 ± 1.4	23.7 ± 0.7	23.3 ± 1.1	23.0 ± 0.7	23.2 ± 0.5	23.1 ± 0.6
DM duration (y)		13.3 ± 9.8	15.4 ± 10.2	17.7 ± 9.1	13.5 ± 6.1	18.3 ± 16.1
HbA1c (%)	5.7 ± 0.3	7.4 ± 1.8	7.6 ± 1.6	7.7 ± 1.4	7.9 ± 0.7	8.1 ± 0.5
Triglycerides (mg/dl)	92.7 ± 40.9	144.3 ± 49.2	156.8 ± 73.8	153.1 ± 18.9	165.7 ± 75.2	122.6 ± 28.9
HDL cholesterol (mg/dl)	63.1 ± 16.0	48.1 ± 10.9	44.9 ± 10.5	43.4 ± 9.6	40.0 ± 5.5	42.6 ± 7.1
LDL cholesterol (mg/dl)	139.5 ± 24.2	103.6 ± 29.6	86.8 ± 31.1	87.8 ± 32.5	101.0 ± 25.4	122.6 ± 28.9
Systolic pressure (mmHg)	135.4 ± 14.4	136.1 ± 16.6	142.7 ± 15.6	153.1 ± 18.9	158.6 ± 25.2	153.7 ± 21.9
Diastolic pressure (mmHg)	77.4 ± 6.8	79.8 ± 9.6	77.8 ± 11.4	80.1 ± 11.8	78.7 ± 8.6	76.7 ± 6.4
Scan quality	95 ± 9.9	94.8 ± 4.1	91.8 ± 4.4	93.5 ± 4.6	94.7 ± 2.6	88.1 ± 5.9
Segmentation accuracy (%)	87.3	75.5	88.9	81.7	85.7	71.4
DME (No. eyes)			4	29	10	5
Smoker / non-smoker (No. eyes)	4 / 67	14 / 35	9 / 18	13 / 47	1 / 13	3 / 4
No (eyes)	71	49	27	60	14	7

DR: Diabetic retinopathy; NPDR: non-proliferative diabetic retinopathy; PDR: proliferative diabetic retinopathy; y: years; BCVA: best-corrected visual acuity; IOP: intraocular pressure; DM: diabetes mellitus; HbA1c: glycosylated haemoglobin; HDL: high-density lipoprotein; LDL: low-density lipoprotein DME: diabetic macular edema; No: number.

Overall, the groups did not differ significantly (p>0.05) in age, spherical equivalent, axial length or intraocular pressure. The difference in BCVA (decimal) was statistically significant between the healthy group and the moderate, severe, and PDR groups (ANOVA, p<0.001); between the T2D without DR and the moderate, severe, and PDR groups (ANOVA, p = 0.004, p = 0.001, p = 0.01, respectively); and between the mild and severe NPDR groups (ANOVA, p = 0.019). Triglycerides were lower in the healthy group than in the T2D groups, except the PDR group, while high-density lipoprotein (HDL) cholesterol and low-density lipoprotein (LDL) cholesterol was higher in the healthy group compared with the T2D groups (ANOVA for HDL cholesterol: p<0.001 for all comparisons, except between the healthy and PDR groups [p = 0.003]; ANOVA for LDL cholesterol: p<0.001, except between the healthy and severe NPDR and PDR groups, p = 0.004 and p = 0.007, respectively). Systolic pressure was lower in the healthy and T2D without DR groups than in the moderate and sever NPDR groups (p<0.009), while diastolic pressure was similar between the groups.

The HbA1c levels and disease duration between the different T2D groups did not differ significantly. The scan quality was significantly lower (p = 0.006) in the PDR group compared with the healthy group and between the T2D without DR and PDR groups (p = 0.012). The accuracy of the automatic segmentation did not differ significantly between the healthy controls and T2D patients or the healthy controls and the DME group (p = 0.47 and p = 0.36, respectively).

### Choroidal thickness measurements

In 10 of the 11 choroidal measurements (SF, T1, T2, T3, T4, T5, N1, N2, N3, and N4), significant differences (p<0.05) were detected between the healthy and T2D groups (Table 2).

**Table 2:** Choroidal thickness measurements in healthy participants and diabetic patients.

	Healthy		T2D group		
	Mean (μm)	SD	Mean (μm)	SD	p
SF	228.1	78.8	189.4	68.9	<0.001*
N1	225.6	81.1	186.9	70.0	<0.001*
N2	213.8	82.5	177.8	72.3	0.001*
N3	194.6	86.6	164.1	73.3	0.007*
N4	168.8	85.0	146.5	71.0	0.04*
N5	146.7	79.3	129.7	68.1	0.10
T1	225.5	74.1	187.4	64.7	<0.001*
T2	221.2	72.0	185.3	66.2	<0.001*
T3	219.3	72.3	179.8	64.5	<0.001*
T4	214.6	68.3	174	62.8	<0.001*
T5	211.2	67.5	170.6	62.9	<0.001*

T2D: Type 2 diabetes; SD: standard deviation; SF: subfoveal; N: nasal position; T: temporal position.

*t test (p<0.05)

Overall, T2D patients presented with a thinner choroid than healthy participants (mean SF thickness was 228.1±78.8 μm in healthy controls and 189.4±68.9 μm in T2D patients; p<0.001). In both groups, measurements revealed a similar pattern: the choroid was thickest in the SF location, followed by temporal and nasal measurements close to the SF area (T1, T2, N1, and N2). The choroid was thinner in the temporal and nasal measurements far away from the SF area (T3, T4, T5, and N3) and the thinnest measurements were in the nasal choroid near the optic disc (N4 and N5).

Table 3 shows the mean choroidal thickness (and standard deviation) of each of the 11 measurements obtained in control group and each group of T2D patients. No differences (p>0.05) were detected between the healthy group and the no DR, mild NPDR, severe NPDR, or PDR groups, respectively. In 8 of 11 choroidal measurements (SF, N1, N2, T1, T2, T3, T4, and T5), significant differences (p<0.05) were detected between the moderate NDPR and healthy groups. Mean SF thickness was 228.1±78.8 μm in healthy controls and 173.7±68.4 μm in moderate NPDR patients (p = 0.003).

**Table 3:** Choroidal thickness measurements in healthy participants and each T2D group.

	Healthy		No DR		Mild NPDR		Moderate NPDR		Severe NPDR		PDR	
	Mean (μm)	SD	Mean (μm)	SD	Mean (μm)	SD	Mean (μm)	SD	Mean (μm)	SD	Mean (μm)	SD
SF	228.1	78.8	191.1	72.7	210.2	73.1	173.7*	68.4	210.7	47.2	188.8	42.4
N1	225.6	81.1	186.9	73.6	210.2	78.4	173.8*	68.6	202.3	50.6	179.8	29.4
N2	213.8	82.5	174.5	74.9	199.6	81.7	168.3*	72.3	191.6	53.2	170.3	30.9
N3	194.6	86.6	160.7	71.3	189.6	93.1	153.2	69.8	175.9	56.8	160.8	44.4
N4	168.8	85.0	145.5	65.1	167.2	93.9	136.3	68.8	155.3	56.1	143.3	50.7
N5	146.7	79.3	130.9	61.5	144.7	89.8	123.5	68.9	128.7	50.1	118.0	39.4
T1	225.5	74.1	188.1	66.4	205.4	63.1	174.0*	69.0	210.5	37.4	182.0	43.9
T2	221.2	72.0	181.4	66.9	200.2	57.4	175.3*	72.6	213.1	52.6	185.4	48.6
T3	219.3	72.3	175.3	64.7	198.3	58.8	170.2*	68.9	203.7	47.5	174.1	65.3
T4	214.6	68.3	170.9	64.7	184.1	61,6	167.5*	66.6	198.1	43.9	164.1	50.0
T5	211.2	67.5	169.5	67.2	176.1	61.1	164.1*	65.5	189.4	44.5	176	52.3

T2D: Type 2 diabetes; SD: standard deviation; SF: subfoveal; N: nasal position; T: temporal position.

*ANOVA, p<0.05 (statistical difference compared to healthy group).

Within the T2D group, we detected no differences (p>0.05) in the choroidal thickness between DME and non-DME patients. When comparing healthy and DME patients (Table 4), however, significant differences (p<0.05) were detected in 9 of 11 measurements (SF, N1, N2, N3, T1, T2, T3, T4, and T5). Mean SF thickness was 228.1±78.8 μm in healthy controls and 183.5±72.9 μm in DME patients (p = 0.002).

In the total sample, HbA1c correlated with SF (r = -0.138; p = 0.039) and N1 (r = -0.146; p = 0.029) choroidal thickness. DM duration had mild correlations with choroidal thickness at SF (r = -0.173; p = 0.030), N3 (r = -0.160; p = 0.046), N4 (r = -0.168; p = 0.036), N5 (r = -0.164; p = 0.041), T1 (r = -0.165; p = 0.040), T2 (r = -0.166; p = 0.039).

**Table 4:** Choroidal thickness measurements in healthy participants and DME patients.

	Healthy		DME patients		
	Mean (μm)	SD	Mean (μm)	SD	p
SF	228.1	78.8	183.5	72.9	0.002*
N1	225.6	81.1	179.4	72.5	0.002*
N2	213.8	82.5	174.6	74.4	0.009*
N3	194.6	86.6	160.2	72.5	0.025*
N4	168.8	85.0	141.5	67.6	0.065
N5	146.7	79.3	122.0	64.1	0.075
T1	225.5	74.1	184.5	68.9	0.003*
T2	221.2	72.0	188.1	69.4	0.014*
T3	219.3	72.3	181.1	68.3	0.005*
T4	214.6	68.3	173.7	62.7	0.001*
T5	211.2	67.5	167.1	60.2	<0.001*

DME: diabetic macular edema; SD: standard deviation; SF: subfoveal; N: nasal position; T: temporal position.

*t test (p<0.05)

In the DME group, mean HbA1c was 7.6±1.1% and DM duration was 14.9±8.6 years. There was a moderate correlation between choroidal thickness in all measurements and HbA1c levels (SF: r = 0.342; p = 0.017). The strongest correlation was observed for choroidal thickness at N5 (r = 0.436; p<0.001). No significant correlation was detected between choroidal thickness and DM duration in DME patients.

## Discussion

SS-OCT uses a longer-wavelength light source than spectral domain OCT, which allows deeper penetration in the choroid than enhanced-depth imaging (EDI) spectral domain OCT and provides more accurate segmentation of the sclero-choroid interface [12,16]. To the best of our knowledge, this is the first study using SS-OCT to measure choroidal thickness in the SF area and at five different nasal and temporal choroidal points in study-naïve T2D patients with different stages of DR presenting with or without DME compared with healthy participants.

Our findings revealed significant thinning in the SF, temporal, and nasal choroidal regions between healthy participants and T2D patients, between healthy participants and moderate NPDR patients, and between healthy participants and DME patients. Ruiz-Medrano et al. demonstrated that choroidal thickness decreases 10–15 μm with each decade of age [15]. In our study, the sample included elderly participants with a mean age of 67.6 years and the groups were well balanced with respect to age. Moreover, diurnal variations in choroidal thickness have been described (maximum peak at morning and progressive decrease throughout the day), with a mean difference of 33.7 μm [3,16–18]. To avoid fluctuations due to time of day, all SS-OCT scans were performed between 4:00 pm and 7:00 pm. Selection criteria included a spherical equivalent ≤6 D and/or cylinder ≤2 D, and an AL ≤26 mm because choroid thickness is associated with AL [19–21].

SF, temporal and nasal choroidal thicknesses were reduced in T2D patients compared to healthy controls. The differences were larger in the SF and temporal regions than in the nasal regions. Differences were observed in 10 of 11 choroidal measurements (5 temporal measurements, SF, and 4 nasal measurements). We detected no significant differences at 2500 μm nasal from the fovea (N5). This may be because the nasal choroid is thinnest near the optic disc, whereas the thickness increases nearer to the fovea [22,23]. Furthermore, we observed mild inverse correlations between HbA1c and central choroidal thicknesses (SF and N1) in the whole population. Though, in the DME group moderate correlations were found between choroidal thickness in all measurements and HbA1c levels. Thus, in DME patients, the increased retinal thickness was related to the increased choroidal thickness. It is likely that in T2D patients with DME, the inflammation contributes to increase both, the retinal and choroidal thicknesses.

Regatieri et al. studied choroidal thickness at the same 11 points using SD-OCT [23]. They included 11 NPDR, 18 DME, and 20 treated PDR patients, and found no significant difference between healthy (24 eyes) and NPDR patients. Although the choroid thickness tended to be lower in the NPDR group than in the healthy group in their study, the sample was too small to detect a significant difference. Their obtained values and the observed pattern in healthy controls are very similar to our results; i.e., the choroid was thicker in the SF region and in temporal and nasal areas near the fovea, and thinner further away from the fovea. The choroid thickness was thinnest nasally near the optic disc.

Querques et al. measured choroidal thickness using SD-OCT [24]. They observed that mean SF choroidal thickness and choroidal thickness at 1.5 mm and 3 mm temporal, nasal, superior, and inferior to the fovea were significantly reduced in NPDR patients without DME compared to the control group. In contrast to our findings, they also found significant differences in the choroidal thickness between T2D patients and the control group near the optic disc. Notably, the mean thickness values in their healthy group were surprisingly greater (for example SF thickness, 309.8±58.5 μm) than in our study (SF thickness, 228.1±78.8 μm) even though both samples were of similar age. Differences in the OCT device used, the retrospective nature of their study, or differences in the AL are likely responsible for these large differences.

Regarding choroidal thickness stratified by ETDRS grading, significant differences were detected in 8 of 11 choroidal measurements between the moderate NPDR and healthy groups (5 temporal measurements, SF, and 2 nasal measurements nearest to the fovea). The thickness pattern was similar in each sub-group and the values were thinner than in the healthy group, but no significant differences were detected among no DR, mild, and severe NPDR, and PDR groups, which may be due to the small number of eyes in these sub-groups compared to that in the moderate NPDR group, which included 60 eyes.

Kim et al. evaluated SF and choroidal thickness at 1500 μm superior, inferior, nasal, and temporal to the fovea [22]. In contrast to previous reports, they found that SF choroidal thickness in PDR was thicker than that in eyes with no DR, or with mild/moderate and severe NPDR. Compared with healthy controls, however, SF and temporal, nasal, superior, and inferior choroidal thickness were slightly decreased in T2D eyes with no DR or with earlier stage NPDR (mild/moderate), although the differences were not statistically significant.

Esmaeelpour et al. evaluated choroidal thickness in 63 T2D eyes [25]. Their choroidal maps showed that SF choroidal thickness was smaller in NDPR patients than in healthy controls. Consistent with our findings, they found a decrease in SF choroidal thickness between a no-DR group, T2D patients with a microaneurysm, and T2D patients with exudates compared to healthy controls. In our study, significant differences were found in 9 of 11 measurements of the choroid (5 temporal, SF, and 3 nasal measurements) in DME patients (48 eyes), showing a thinner choroidal thickness than healthy controls. In accordance with our findings, Regatieri et al. reported the same significant differences at the same locations in the choroid between DME and healthy patients [23]. Querques et al. also reported a reduced choroidal thickness at the SF and at 1.5-mm and 3-mm nasal, temporal, superior, and inferior in DME patients compared to healthy controls [24]. Esmaeelpour et al. [25] and Adhi et al. [26] also found that the SF choroid was thinner in DME patients compared with healthy eyes. Contrary to these results, Kim et al. found that SF choroidal thickness was related to increased severity of DR (from no DR to proliferative DR) and with the presence of DME, particularly in those eyes with serous retinal detachment [22]. Nevertheless, it remains unclear whether the greater choroidal thickness could be related to local inflammation or if this discrepancy is due to differences in study design and patient profiles.

In our study, we detected no significant differences in HbA1c levels in the T2D groups, but there was a moderate correlation between choroidal thickness and HbA1c levels in DME patients (SF r = 0.342, p = 0.017). In contrast to our findings, Kim et al. found a significant difference in the HbA1c levels between DR groups [22]. They also found a significant correlation between HbA1c and SF choroidal thickness (r = 0.252, p<0.05).

The strengths of this study include the large sample size with study-naïve T2D elderly patients, grading retinopathy, and the use of SS-OCT with several measurements at different points in the choroid. The main limitations are the relatively small sample size for the severe and proliferative T2D groups and the lack of totally automatic segmentation software. Manual corrections were made to avoid mis-segmentations, making the technique semi-automatic.

The relationship between DR and diabetic choroidopathy is not clearly defined in the literature [5]. The choroidal layer supplies oxygen and nutrients to the outer retina. Any change or damage with thinning to this tissue may affect the overlying retina, causing hypoxia and leading to the appearance of DR lesions or the progression of existing retinopathy. However, whether the thinning of the choroid is prior to the appearance of DR lesions or if the DR lesions are associated with the reduction of the choroidal thickness remains unknown. Therefore, expanding our knowledge of the pathophysiologic mechanisms involved in DR, including those affecting the choroid, may help clinicians to better understand the course of the disease and optimize the management of DR based on tailored interventions.

In conclusion, choroidal thickness was significantly reduced in T2D patients compared to age-matched controls. Further studies are needed to clarify the effect of diabetes on the choroid and the overlying retina.

## Supporting information

S1 Database Database for choroidal measurements. (XLSX)
